# Adherent Use of Digital Health Trackers Is Associated with Weight Loss

**DOI:** 10.1371/journal.pone.0152504

**Published:** 2016-04-06

**Authors:** Arya Pourzanjani, Tom Quisel, Luca Foschini

**Affiliations:** Evidation Health, Menlo Park, CA, United States of America; Texas A&M University, UNITED STATES

## Abstract

We study the association between weight fluctuation and activity tracking in an on-line population of thousands of individuals using digital health trackers (1,749 ≤ *N* ≤ 14,411, depending on the activity tracker considered) with millions of recorded activities (119,292 ≤ *N* ≤ 2,221,382) over the years 2013–2015. In a first between-subject analysis, we found a positive association between activity tracking frequency and weight loss. Users who log food with moderate frequency lost an additional 0.63% (CI [0.55, 0.72]; *p* < .001) of their body weight per month relative to low frequency loggers. Frequent workout loggers lost an additional 0.38% (CI [0.20, 0.56]; *p* < .001) and frequent weight loggers lost an additional 0.40% (CI [0.33, 0.47]; *p* < .001) as compared to infrequent loggers. In a subsequent within-subject analysis on a subset of the population (799 ≤ *N* ≤ 6,052) with sufficient longitudinal data, we used fixed effect models to explore the temporal relationship between a change in tracking adherence and weight change. We found that for the same individual, weight loss is significantly higher during periods of high adherence to tracking vs. periods of low adherence: +2.74% of body weight lost per month (CI [2.68, 2.81]; *p* < .001) during adherent weight tracking, +1.35% per month (CI [1.26, 1.43]; *p* < .001) during adherent food tracking, and +0.60% per month (CI [0.44, 0.76]; *p* < .001) during adherent workout tracking. The findings suggest that adherence to activity tracking can be utilized as a convenient real-time predictor of weight fluctuations, enabling large-scale, personalized intervention strategies.

## Introduction

Body weight abnormalities are estimated to cost 21% ($190.2 billion) of annual medical spending in the United States [1, 2]. Even if a few policies and interventions [3–5] have been successful in slowing down the prevalence of obesity in recent years [6] the overall picture is still daunting, as a recent study reported that the adult obesity rate in the U.S. increased by more than two percent from 25.5% to 27.7% in just six years [7]. It is well known that an increased awareness of factors affecting weight helps individuals better manage their weight. Simple weight monitoring has been shown to be linked with weight loss both in observational studies [8–10], and randomized control trials [11–13]. In addition, studies have shown that monitoring eating habits [14, 15] and exercise levels [16] are also conducive of weight loss.

The extent of the association between monitoring frequency and amount of weight loss has also been investigated, although less thoroughly. VanWormer [17] ranked 12 weighing frequency studies in terms of methodological quality finding only one (Wing et al. [18]) to be worth an A rating. Burke et al. [19] found the a positive correlation between weight loss and the frequency at which individual monitor weight-related activities. Studies linking monitoring adherence and weight loss usually consider settings in which weight frequency is self-reported retrospectively [20] or it is summarized to a single frequency value that fails to capture temporal variation in self-weighing activity [21]. An exception can be found in the very recent study by Helander et al. [22] that investigates how the temporal variation in frequency of weight monitoring affects changes in body weight, showing that breaks in weight self-monitoring longer than 6 days are associated with weight gain. Their study, however, considered a very small (*N* = 40) and biased (all Scandinavian, participating in a health promotion program) population and does not analyze tracked activities other than weight monitoring. In the conclusion of their paper, Helander et al. note that it is unclear whether and to what extent their findings generalize to a larger population independently recording multiple activities outside the context of a predetermined program, and envisioned that their own analysis could be repeated on data sourced from a large on-line population of individuals reporting their weight using a connected Wi-Fi scale.

In this paper we analyze millions of recordings automatically collected through digital health trackers for monitoring weight, exercise, and food intake, and surface temporal patterns of interaction with trackers that are associated with weight change over time. The population studied is orders of magnitude larger than what previous studies have considered, consisting of several thousands of individuals observed over more than two years. The inferences we present are derived from real-world usage data and ultimately link *observed* tracking behavior in a large-scale and distributed setting to weight loss. In addition, the population studied consists of individuals who have not expressed an explicit interest in losing or controlling their weight. The temporal links we uncover between tracking behavior and weight change are on the timescale of days, allowing for prompt intervention strategies.

## Methods

### Data

The population under analysis is a subset of the users of a commercial reward platform for aggregating healthy activities (AchieveMint, powered by Evidation Health, Menlo Park, CA). On the platform, users link their activity trackers (e.g., Fitbit pedometers and Wi-Fi scales, Jawbone tracker, etc.) and apps (e.g., MyFitnessPal, RunKeeper) by authorizing their data to be relayed to their reward platform account. Users can connect multiple apps/trackers to the platform. For example, some users might have connected both a Wi-Fi scale and a food journaling app, while others may have connected a pedometer and a workout-tracking app. For every new activity reported through their third-party apps and devices, users earn points. Points are redeemable for cash rewards: after a user has achieved 1,000 points, they will earn $1.00. Users receive a check for every $25.00 earned. This study analyzes weight measurements via a Wi-Fi scale, food logging via an app, and workout logging (defined as a bike ride, run, or minutes at the gym reported via an app or fitness tracker). The platform rewards each new weight measurement with 10 points, each entry of food logging with 30 points, and each recording of a workout with 10 points.

Consent for participation in this study was obtained electronically by accepting a terms of service contract for the reward platform. Obtaining an additional written consent would not be feasible as some of the subjects are no longer a member of the platform and are unreachable. The study was approved by Solutions Institutional Review Board and determined to be exempt from the OHRP’s Regulations for the Protection of Human Subjects (45 CFR 46) under the following categories: Category 4–Research involving the collection or study of existing data, documents, records, pathological specimens, or diagnostic specimens, if these sources are publicly available or if the information is recorded by the investigator in such a manner that subjects cannot be identified, directly or through identifiers linked to the subjects.

#### Inclusion/Exclusion Criteria

**Fig 1:**
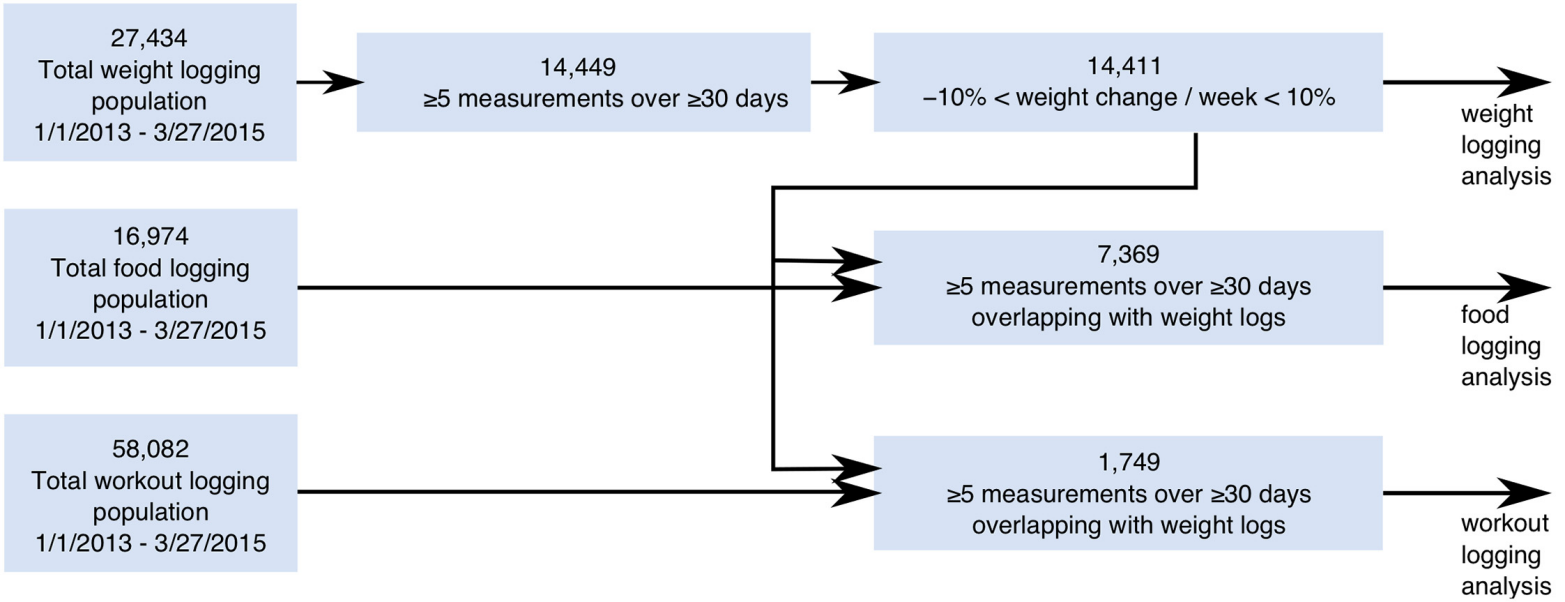
Flowchart of inclusion/exclusion criteria for the primary analysis.

We included in the primary analysis all users from the reward platform population who have a Wi-Fi scale connected and at least five weight measurements logged over at least a 30-day period between January 1st, 2013 and March 27th, 2015. Users reporting an average monthly weight change of 10% or more were excluded from the analysis. This threshold excludes 38 users (0.3%) and appears from the data to be a reasonable cutoff for misreported values, such as a different person using the scale. For the food and workout analyses, users had to meet the additional requirement of having at least five food (resp. workout) recordings during at least a 30-day period. Finally, to ensure overlap between measurement periods, included users were required to have at least five food (resp. workout) recordings within their first and last weight measurements, and conversely, five weight measurements between the first and last food (resp. workout) measurements. Fig 1 depicts the selection process. Table 1 show summary statistics for users included in the primary analysis. The duration of monitoring reported is the number of weeks between the first and the last measurements observed for the various activities.

**Table 1:** Population characteristics for the primary analysis: mean (standard deviation) [number not reported].

	Weight	Food	Workout
**Individuals**	14,411	7,369	1,749
**Gender (Male)**	1,883 [5, 702]	980 [1, 915]	443 [114]
**Age (Years)**	38 (9) [6, 449]	38 (9) [2, 351]	38 (9) [149]
**BMI (kg**/m^2^)	31 (8) [7, 280]	31 (7) [2, 809]	29 (7) [333]
**Weight (kg)**	87 (21)	87 (22)	84 (19)
**Weight Change (%/month)**	-0.4 (1.3)	-0.3 (1.2)	-0.4 (1.1)
**Duration of Monitoring (weeks)**	41 (33)	26 (18)	35 (23)
**Number of Logs**	74 (141)	289 (354)	63 (98)
**Number of Logs Per Week**	2.1 (2.9)	11.8 (10.0)	2.0 (2.6)

We note that among the users who reported BMI, the distribution of BMI was similar to the United States distribution, with a mode of 25. The duration of monitoring reported is the number of weeks between the first and the last measurements observed.

**Fig 2:**
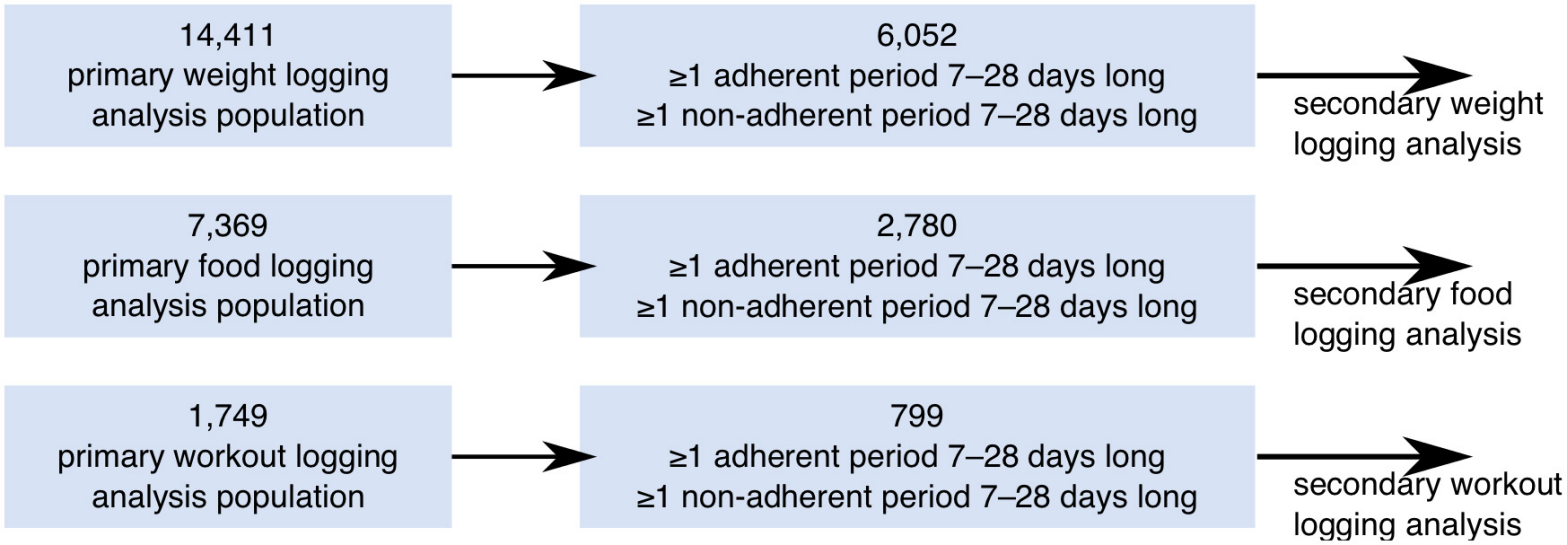
Flowchart of inclusion/exclusion criteria for the secondary analysis.

We included in the secondary analysis all the users of the primary analysis that met additional criteria on the longitude of their data. Specifically, included users had at least one adherent and one non-adherent tracking period, where a period is defined to be adherent if there are no gaps longer than 4 days between any two consecutive measurements. The maximum gap length of 4 days was chosen to ensure that users did not take too long a break in reporting during adherent periods, following Helander et al. [22]. Both adherent and non-adherent periods were required to be between 7 and 28 days in length to be counted towards the inclusion criteria. We required that periods be at least a week long to allow potential benefits associated with the adherent period to be detected [23], and not too long to prevent extended periods of missing data due to technical reasons to be counted as non-adherent periods. Fig 2 depicts the selection process. The chosen gap and period lengths are further validated in the analysis section. Summary statistics for users included in the secondary analysis are reported in Table 2. Table 3 describes period count and length for users in the secondary analysis.

**Table 2:** Population characteristics for subjects in the secondary analysis: mean (standard deviation) [number not reported].

	Weight	Food	Workout
**Individuals**	6,052	2,780	799
**Gender (Male)**	1010 [2, 126]	360 [540]	207 [48]
**Age (Years)**	39 (9) [2, 434]	37 (9) [684]	38 (9) [63]
**BMI (kg**/m^2^)	30 (7) [2, 746]	31 (7) [894]	29 (7) [125]

**Table 3:** Distribution of number of periods per user and median period length per user for all three log types in the secondary analysis.

	Min	Q25	Q50	Q75	Max
**Number of Weight logging Periods**	2	3	5	9	50
**User’s Median Weight logging Period Length (days)**	7.0	10.5	12.2	14.5	28.0
**Number of Food logging Periods**	2	3	4	7	28
**User’s Median Food logging Period Length (days)**	7.0	10.0	12.0	15.0	28.0
**Number of Workout logging Periods**	2	3	5	8	43
**User’s Median Workout logging Period Length (days)**	7.0	10.2	12.0	14.5	27.0

To calculate period length per user we take the median of period length of each user and compute quantiles of the entire population from this result. Note that these values are measured after excluding periods of unacceptable length as described in the secondary analysis.

### Analysis

The primary analysis measured the association between weight loss and tracking frequency for users of each gender in our population while controlling for age and duration of tracking. In the secondary analysis we studied the association of periods of high and low tracking adherence with weight change while controlling for inter-user variation and period length.

All significances reported use two-tailed t-tests with a significance level of *α* = 0.05. Analysis was performed in R 3.2.3 [24], and used the plm package [25] for panel regression in the secondary analysis.

#### Between-subject Analysis

In our primary analysis we used separate linear regressions to model weight change for each of three groups: all users, female users, and male users, and for each of the three kinds of activity tracked: weight, food, and workouts, for a total of 9 regressions. The population sample skews female, and performing a separate regression for each gender ensures that results generalize to males as well. We included age and tracking duration (the total number of weeks that the user tracked the activity) in the model to control for any confounding effects these variables may have. The regression equation is as follows:

$${\text{Δ}}_{\text{weight}}\;=\;\beta_{0}+\beta_{1}A+\beta_{2}F+\beta_{3}D+\beta_{4}\text{FD}+\epsilon$$

The outcome variable, **Δ**_**weight**_ is the average percent weight change per month for each user over the period during which the activity was tracked. We use relative weight as a way to control for baseline weight and eliminate heteroskedasticity of absolute changes in weight. This approach is also discussed in Helander et al. [22], where they reported that controlling for baseline weight did not alter their results. To compute relative weight for a user, each weight measurement in the time series is divided by the earliest measurement for that user.

**A** is the age of each user, included to control for age. *β*_0_ and **ϵ** are the intercept and error terms, respectively. **F** is the mean-centered logarithm of the average number of recordings of the activity per week for each user. **D** is the mean-centered logarithm of the duration (in weeks) over which the user tracked the activity.

We include the interaction term **FD** in addition to **D** to better assess the association between **Δ**_**weight**_ and **F**. We expected that monthly weight change would decrease as tracking duration increased, and including **D** and **FD** allows us to asses the range of values of **D** for which the association between **Δ**_**weight**_ and **F** is significant. By mean-centering **F** and **D**, their coefficients are easier to interpret in light of the interaction term. The coefficient for **F** can be interpreted as the simple slope for the linear relationship between **F** and **Δ**_**weight**_ while holding **D** constant at its mean and controlling for all other covariates. The analogous statement is true for the coefficient for **D**. The log transforms for **F** and **D** were used to convert heavy-tailed variables bounded at 0 to near-normally distributed variables that are more suitable for inclusion in a linear model.

#### Within-subject Analysis and Adherent Tracking

In the secondary analysis, we analyzed the association of tracking adherence and weight change while controlling for differences among users and length of adherent/non-adherent period. To this end, rather than computing a single frequency value per user over the time when the activity is tracked, we broke up the tracking period into “adherent” periods and “non-adherent” periods. An adherent recording period is a time interval containing recordings with no gap longer than 4 days between consecutive recordings, with the first and last recordings in the interval separated from the rest of the sequence by gaps longer than 4 days. Non-adherent periods are the periods between adherent periods and have gap lengths that are always longer than 4 days. We only considered periods with length between 7 and 28 days. We then used linear interpolation to impute each user’s weight at any point in time, and defined weight change over the period to be the difference between the imputed weight at the end of the period and the imputed weight at the start of the period. We assessed the robustness of our findings to the definition of adherent and non-adherent periods by repeating the analysis for different choices of the maximum allowed gap between consecutive recordings during adherent periods and of the minimum and maximum period lengths allowed.

To ensure that findings were also applicable to males despite their under-representation, we performed the secondary analysis on both genders separately, as well as on the full population. We modeled the relationship between periods and weight change using fixed-effects and random-effects models. Both kinds of models gave the same result, so only the fixed-effect model is described and reported.

We included in the model an indicator of whether a period was adherent or non-adherent as a fixed effect. This allowed us to assess our hypothesis that users lose more weight during adherent periods. Individual differences in weight change during periods were accounted for by including the user as a fixed effect. Possible confounding effects of period length were controlled for by including period length in the regression. The model is summarized by the following regression equation:

$${\text{Δ}}_{\text{ij}}^{\text{weight}}\;=\;\alpha_{i}+\beta_{1}N_{\text{ij}}+\beta_{2}L_{\text{ij}}+u_{ij}$$

In this equation, ${\text{Δ}}_{\text{ij}}^{\text{weight}}$ is the monthly relative weight change during the *j*’th period for user *i*. *α*_*i*_ is the fixed effect included for each user, **N**_**ij**_ is a 0–1 indicator for whether the user was non-adherent during that period, **L**_**ij**_ is the length of the period in days, and *u*_*ij*_ is the error term.

The coefficient for **N**_**ij**_, is the variable of primary interest. It measures the difference in weight change observed during non-adherent periods versus adherent periods.

## Results

### Between-subject Analysis

In the primary analysis, when controlling for age, duration of logging, and interactions, we found a positive association between higher average logging frequency and average weight loss. This positive association was significant for both genders across all activities (food logging, heavy exercise logging, and weight logging). Table 4 summarizes the estimated coefficients in the linear models along with their significance and confidence intervals.

**Table 4:** Primary analysis linear regression coefficient estimates, 95% confidence intervals, and p-values.

	Weight			Food			Workout		
Variable	Coef	CI	P-val	Coef	CI	P-val	Coef	CI	P-val
***All***									
***β***_**0**_	-0.05	[-0.14, 0.04]	.28	-0.15	[-0.27, -0.02]	.02	-0.44	[-0.67, -0.20]	<.001
**A**	-0.00	[-0.01, -0.00]	<.001	-0.00	[-0.01, 0.00]	.08	0.00	[-0.00, 0.01]	.56
**F**	-0.14	[-0.16, -0.12]	<.001	-0.20	[-0.22, -0.17]	<.001	-0.12	[-0.18, -0.07]	<.001
**D**	0.28	[0.25, 0.30]	<.001	0.37	[0.33, 0.41]	<.001	0.35	[0.27, 0.43]	<.001
**FD**	0.08	[0.06, 0.10]	<.001	0.11	[0.07, 0.14]	<.001	0.08	[-0.00, 0.16]	.05
***Female***									
***β***_**0**_	-0.04	[-0.15, 0.06]	.42	-0.06	[-0.20, 0.08]	.42	-0.42	[-0.69, -0.14]	.003
**A**	-0.01	[-0.01, -0.00]	<.001	-0.01	[-0.01, -0.00]	.005	0.00	[-0.01, 0.01]	.92
**F**	-0.15	[-0.17, -0.12]	<.001	-0.19	[-0.22, -0.16]	<.001	-0.10	[-0.17, -0.04]	.002
**D**	0.29	[0.26, 0.32]	<.001	0.38	[0.33, 0.42]	<.001	0.41	[0.32, 0.50]	<.001
**FD**	0.09	[0.06, 0.12]	<.001	0.11	[0.07, 0.15]	<.001	0.07	[-0.03, 0.16]	.19
***Male***									
***β***_**0**_	-0.06	[-0.26, 0.14]	.54	-0.56	[-0.86, -0.25]	<.001	-0.40	[-0.87, 0.06]	.09
**A**	-0.00	[-0.01, 0.00]	.22	0.01	[-0.00, 0.01]	.07	0.00	[-0.01, 0.01]	.52
**F**	-0.13	[-0.17, -0.09]	<.001	-0.23	[-0.29, -0.16]	<.001	-0.19	[-0.29, -0.08]	<.001
**D**	0.23	[0.17, 0.30]	<.001	0.34	[0.24, 0.43]	<.001	0.19	[0.04, 0.34]	.02
**FD**	0.06	[0.01, 0.11]	.01	0.10	[0.01, 0.18]	.03	0.15	[0.00, 0.29]	.05

The coefficients for **F** are of interest in the primary analysis, demonstrating the association between increased tracking frequency and decreased weight gain.

To better interpret effect sizes, in Table 5 we report for each activity the magnitude, significance, and confidence interval of the logging/weight-loss association at the median value of logging duration for a change of logging frequency from low to moderate. To define the moderate logging frequencies, we picked values that have been known to be associated with improved weight control for each activity: once a day for food [26], once a week for weight [22], and three times a week for workout logging [14, 16]. Conversely, for low logging frequencies we picked values that are not expected to be associated with any improvement in weight management. This was achieved by setting the low frequency to be 30x lower than the moderate frequency: once a month for food, once every 30 weeks for weight, and once every 10 weeks for workout logging. Considering the same (30x) relative change in logging frequency from moderate to low for all activities has the additional benefit of making effect sizes comparable across activities.

**Table 5:** Amount of additional weight loss associated with increased logging rate, for each activity.

	Weight	Food	Workout
**Median Logging Duration**	46.1 weeks	25.6 weeks	31.8 weeks
**Example Logging Rate Increase**	1x/30 weeks to 1x/week	1x/month to 1x/day	1x/10 weeks to 3x/week
**Additional Weight Loss (%/month)**	0.40 (CI [0.33, 0.47])	0.63 (CI [0.55, 0.72])	0.38 (CI [0.20, 0.56])
**P-value**	<.001	<.001	<.001

The additional weight loss is in units of percent of body weight per month, and is estimated at the median user logging duration for each activity. The example logging rate increases all correspond to a 30x increase in logging frequency, and they exemplify an increase from a low to a moderate frequency for the specific activity considered.

The food logging analysis shows that users who log food with moderate frequency lost an additional 0.63% (CI [0.55, 0.72]; *p* < .001) of their body weight per month relative to low frequency loggers. Moderate workout loggers lost an additional 0.38% (CI [0.20, 0.56]; *p* < .001), and moderate weight loggers lost an additional 0.40% (CI [0.33, 0.47]; *p* < .001).

As expected, an increased length of the observed period of measurement (logging duration) is associated with a lower weight loss percent per month for all activities and genders. This is a natural consequence of the transitory nature of weight loss, by which a constant amount of weight loss per month cannot be sustained for a prolonged period of time, in turn making longer periods of observations more likely to display lower average relative weight loss per month.

**Fig 3:**
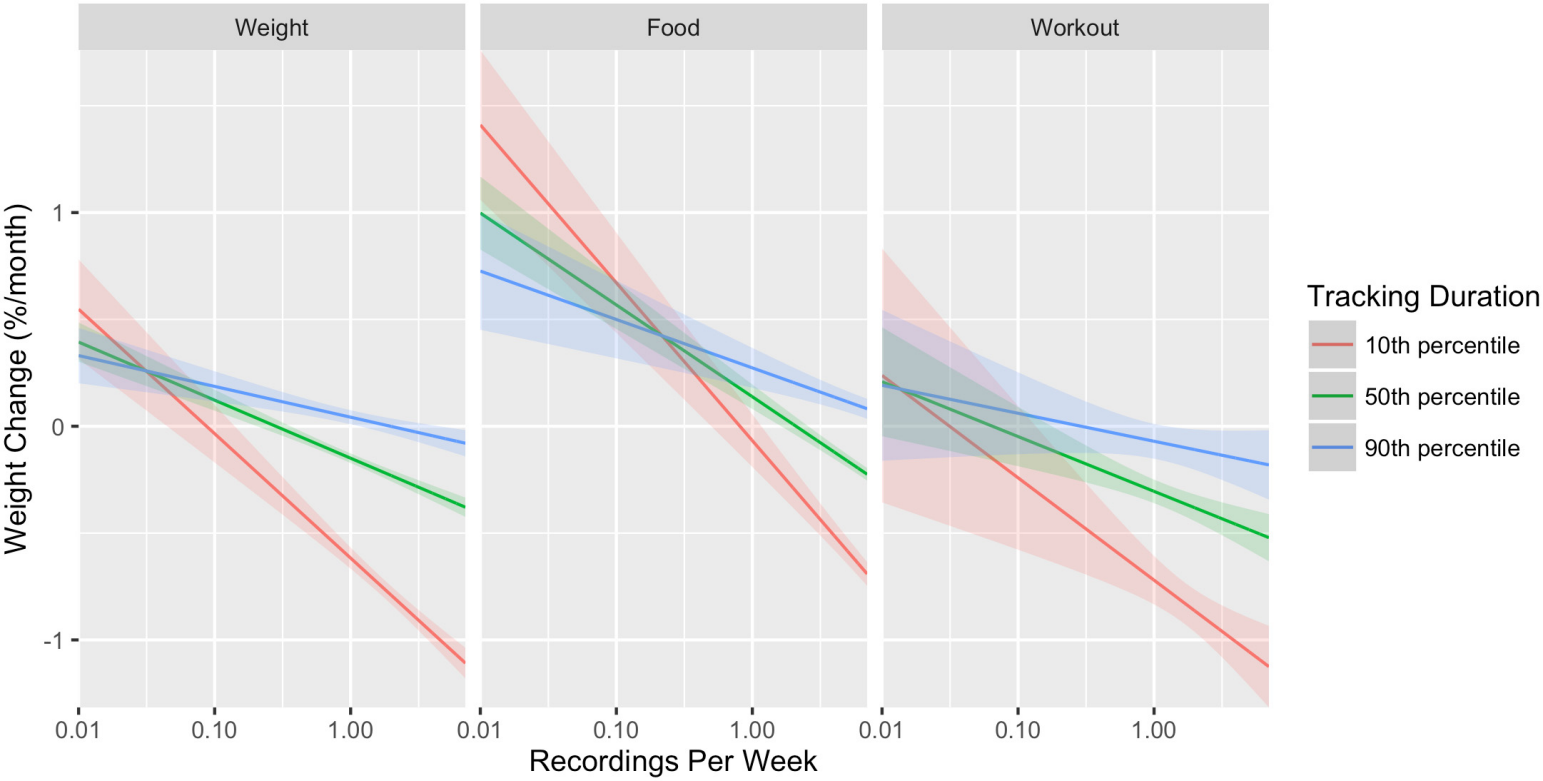
Plot of the modeled association between weight change and activity tracking frequency for both genders, over all activities, and for different monitoring durations. Note that that frequency on the *x* axis is log scaled. The 10, 50, and 90 percentiles of tracking duration were chosen to represent users who monitored their activity for short, medium, and long durations, and to demonstrate how the association between weight change and activity tracking frequency varied with monitoring duration. In general, increased monitoring duration is associated with decreased weight loss per month and a weaker association between tracking frequency and weight change. The confidence bands are 95% confidence intervals. We note that the three lines intersect in every graph at the point where *F* = −*β*_3_/*β*_4_, recalling that *F* is the mean-centered logarithm of recordings per week.

The significant effect of the interaction terms **FD** show that the association between relative weight loss per month and logging duration varies for different logging durations considered, as illustrated in Fig 3. However, the association remains significant for a wide range of values of tracking duration as summarized in Table 6, in which the regions of significance for the association across logging durations for each activity type are reported.

**Table 6:** Regions of significance for the tracking frequency–weight loss association in the primary analysis.

	Weight	Food	Workout
***All***			
**Significant Range of Tracking Duration (weeks)**	4.3–115.3	4.3–76.0	4.3–55.6
**Significant Range of Tracking Duration (percentile)**	0–98	0–99	0–85
***Female***			
**Significant Range of Tracking Duration (weeks)**	4.3–115.3	4.3–76.0	4.3–45.8
**Significant Range of Tracking Duration (percentile)**	0–98	0–99	0–74
***Male***			
**Significant Range of Tracking Duration (weeks)**	4.3–96.1	4.3–72.0	4.3–45.8
**Significant Range of Tracking Duration (percentile)**	0–91	0–98	0–74

Tracking duration values represent the length of the observed recording period for a given activity. The reported ranges (top row) for each activity identify the interval of tracking durations for which the results of the primary analysis summarized in Table 4 remain statistically significant. The regions of significance are wide, as denoted by the correspondent percentile ranges (bottom row), indicating that the associations found are robust. Note that 4.3 weeks corresponds to 1 month, the minimum tracking duration included in the analysis.

### Within-subject Analysis

We found that adherent periods are associated with significantly higher weight loss than non-adherent periods across all activity types for the whole population, as well as for each gender analyzed separately. On average, users lost an additional 2.74% (CI [2.68, 2.81]; *p* < .001) of their body weight per month during adherent periods of weight tracking, 1.35% (CI [1.26, 1.43]; *p* < .001) for food logging, and 0.60% (CI [0.44, 0.76]; *p* < .001) for workout tracking. Coefficients, confidence intervals, and p-values for each of the three models are in Table 7.

**Table 7:** Secondary analysis linear regression coefficient estimates, 95% confidence intervals, and p-values.

	Weight			Food			Workout		
Variable	Coef	CI	P-val	Coef	CI	P-val	Coef	CI	P-val
***All***									
**N**	2.74	[2.68, 2.81]	<.001	1.35	[1.26, 1.43]	<.001	0.60	[0.44, 0.76]	<.001
**L**	0.01	[0.01, 0.02]	<.001	0.01	[0.01, 0.02]	.03	0.00	[-0.01, 0.01]	.88
***Female***									
**N**	2.86	[2.77, 2.95]	<.001	1.29	[1.19, 1.39]	<.001	0.61	[0.42, 0.80]	<.001
**L**	0.02	[0.01, 0.03]	<.001	0.01	[-0.01, 0.02]	.06	0.00	[-0.02, 0.02]	.83
***Male***									
**N**	2.36	[2.21, 2.51]	<.001	1.38	[1.12, 1.63]	<.001	0.54	[0.22, 0.87]	.001
**L**	-0.01	[-0.02, 0.01]	.34	0.01	[-0.02, 0.03]	<.001	0.01	[-0.02, 0.05]	.35

**N** coefficients are in units of %/month and can be interpreted as the difference in relative monthly weight change associated with a switch from an adherent logging period to a non-adherent logging period while controlling for period length. The coefficients are significantly different from zero in all cases, indicating increased weight gain during non-adherent periods.

**Fig 4:**
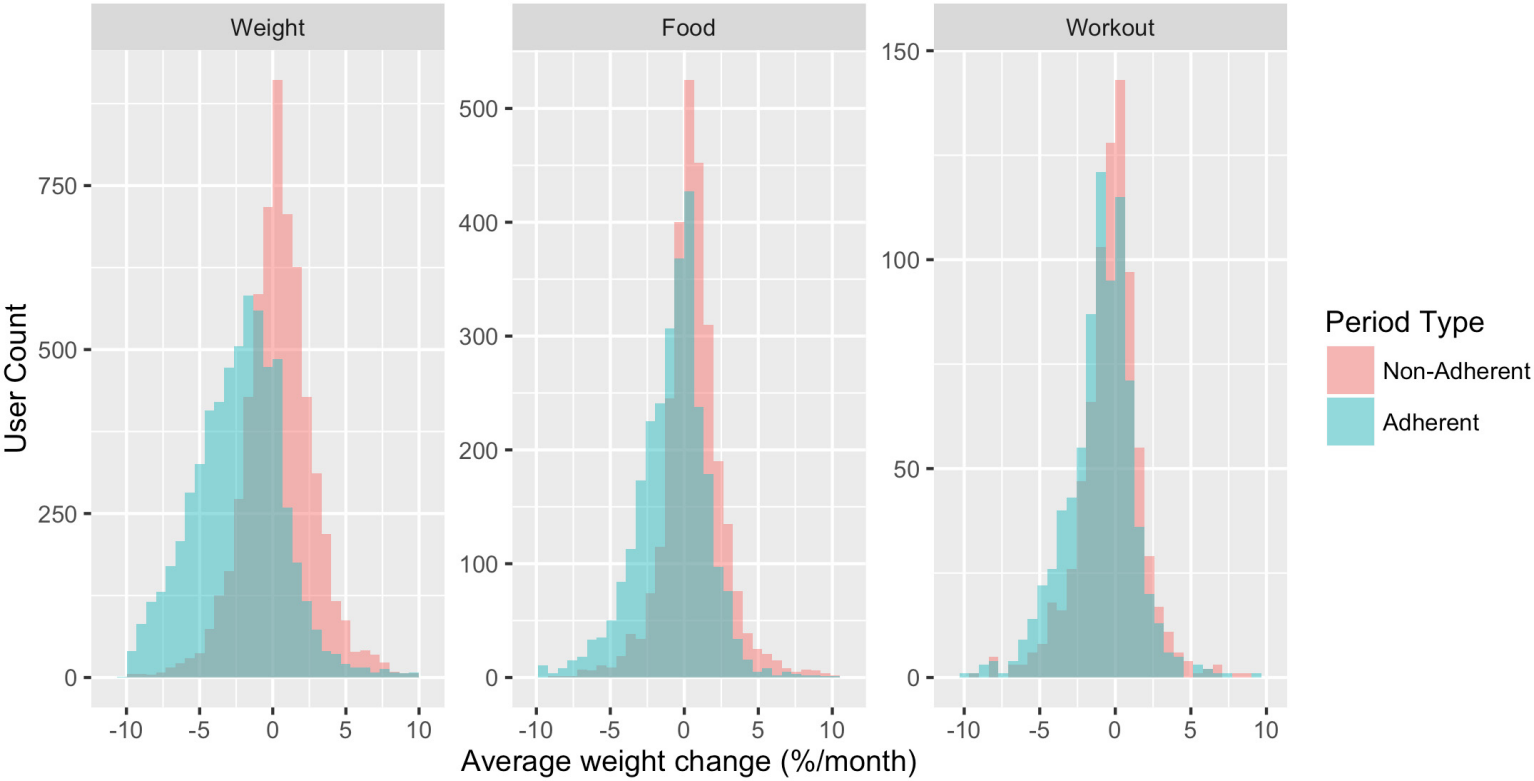
Histogram of average weight change for users during adherent and non-adherent periods of activity tracking. The histograms depict the association between logging adherence and weight change while controlling for all user variation (gender, age, weight, etc.). For each user in the secondary analysis, average weight change is computed first for their adherent tracking periods and then for their non-adherent periods. Both averages are histogrammed for each activity type. The graph shows a positive association between logging adherence and weight loss, as the adherent weight-change distribution is shifted left relative to the non-adherent weight-change distribution.

Histograms of per-user weight change during adherent and non-adherent periods make this association apparent (see Fig 4), and summary statistics for weight change during the adherent and non-adherent periods can be seen in Table 8. The summary table puts the regression results in context. While there is a strong population-wide association between weight loss and adherent tracking, individual rates of weight change during adherent and non-adherent periods display high individual variability.

**Table 8:** Mean per-user weight change during adherent and non-adherent periods in the secondary analysis: mean (standard deviation).

	Weight	Food	Workout
**Adherent period weight change (%/month)**	-2.36 (3.05)	-0.80 (2.40)	-0.98 (2.29)
**Non-adherent period weight change (%/month)**	0.42 (2.39)	0.62 (2.04)	-0.30 (2.07)

Values were computed by first averaging weight change for periods within each user, and then averaging (or computing standard deviation) across all users. Note that weight loss is higher during adherent periods, and that variation from user to user is high relative to the mean.

**Fig 5:**
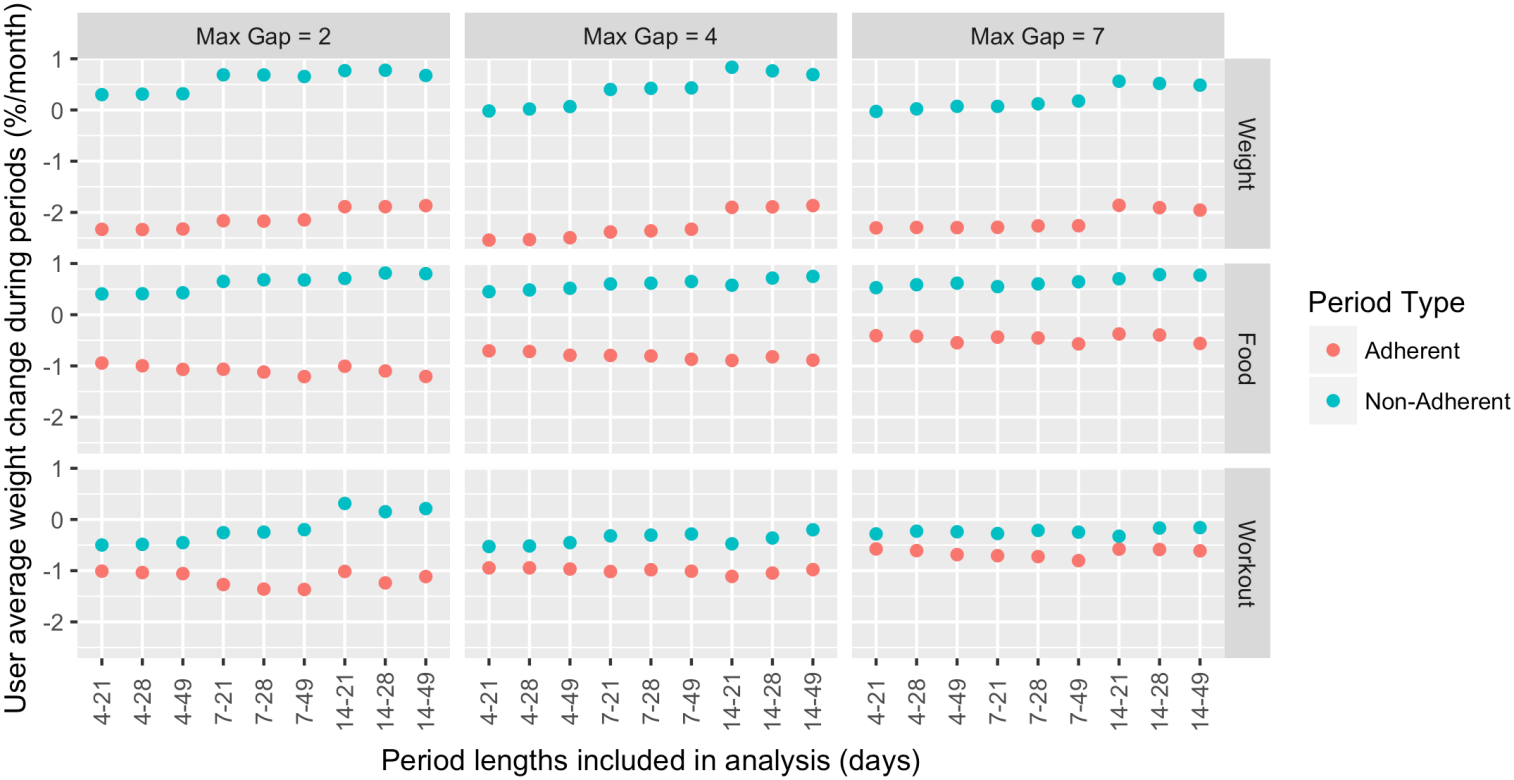
Average weight change during adherent and non-adherent periods for various definitions of adherent period. We observe that non-adherent periods have higher weight loss, regardless of the value of max gap or period length. The secondary analysis was performed with a max gap of 4 days, and including periods of 7–28 days in length.

In Fig 5 we report a sensitivity analysis considering varying maximum gap sizes for adherent periods (4 days in the main analysis) and required minimum and maximum length of periods (7–28 days in the main analysis). We observe that effect sizes remain stable around the parameters used in the main analysis, confirming the intuition that the differential effect on weight loss of adherent vs. non-adherent activity tracking tapers off as the maximum gap size increases. The effects also tend to flatten out as longer range of periods are considered. This is intuitive as the outcome variable considered measures the change of relative weight per unit of time, and high rates of weight change are unlikely to be sustained for long periods of time.

## Discussion

Our analysis shows that adherence to activity tracking is a good predictor of weight change. The positive association between adherence to activity tracking and weight loss is consistent with past studies on weight self-monitoring [18, 22, 27] and food and workout logging [14–16]. To the best of our knowledge, the temporal relationship between adherence to tracking and weight loss surfaced in our secondary analysis had not been explored before for general activity tracking. When restricted to weight self-monitoring only, our secondary analysis confirms previous findings on the directionality of the association [28, 29] and the high individual variability observed [22].

The primary novelty of our analysis lies in its setting, as our sample sizes are unprecedentedly high for studies of this nature that exist in the literature [19] in terms of number individuals (several thousand, depending on the activity) activity logs (several million) and longitude (more than two years). In addition, the population considered was not proactively enrolled in any weight loss programs or experiments, and the analyzed data was sourced by the users’ connected devices without solicitation—a setting that constitutes a major departure from the controlled environment described in previous research. We note that in a distributed setting such as the one considered, the decoupling between performing an activity and tracking it might be substantial (due to missing data, incorrect reports, etc.), therefore establishing a link between the *observed* behavior through the lens of the tracking device and the outcome variable does not immediately follow from previous studies in which observations were sourced from a controlled environment.

One limitation of our analysis is self-selection bias of the population under study. The on-line population considered consist of individuals who elected to buy a tracking device, connect them to the reward platform, and log at least a handful of activities. This indicates that the individuals may be on average more motivated to lose weight. We believe that the extra motivation towards weight loss, however present, should be comparable to that of other populations analyzed in previous studies, the majority of which belonged to weight loss programs or enrolled in experiments explicitly advertised as studying weight loss.

Another limitation to generalizing the findings of the present study is that users were receiving monetary incentives for logging activities and the findings might not hold if incentives were to be removed. We argue that is likely not the case as our inclusion/exclusion criteria selected users who had been on the platform for at least a few months, which is a time horizon beyond which it is known that monetary rewards, much like other external motivation strategies, tend to lose efficacy on habit formation [30–32].

## Conclusion

We presented an analysis of the association between weight loss and temporal patterns of weight self-monitoring, food logging, and workout tracking. Our findings show that users who track activities more frequently on average tend to lose more weight than their peers who track activities less frequently. We also show that the claim still holds when considered longitudinally: when an individual increases their adherence to tracking they are more likely to lose or maintain their weight.

An overarching conclusion of this study is that data collected from activity trackers can inform real-time interventions [33] designed to adapt to users’ heterogeneous natures and performance, and targeted to improve the health outcome monitored. It is worth noting that such interventions need only rely on the predictive power of the observed variable to the target variable, irrespective of any direct casual link. For example, both increased adherence to activity tracking and weight loss could be the reflection of a person’s internal tendency to engage in health-enhancing behavior, a hypothesis known as “healthy user effect” [34]. In the “healthy user effect” hypothesis, no direct causal link between adherence to activity tracking and weight change is present, however effective interventions could still be designed, triggered by an observed drop in activity-tracking adherence, to nudge an individual into the “healthy state” (e.g., by reminding the user of the importance of healthy habits) thus ultimately affecting their weight.

The opportunity to leverage digital health technologies to extend the notion of precision medicine to preventative care [35] becomes especially relevant now that activity trackers are becoming mainstream, with a market size projected to increase to more than $50 billion by 2018 [36, 37]. However, as argued by Patel et al. in a recent opinion paper [38], the successful use and potential health benefits of digital health technologies does not only depend on penetration and adoption, but on the design of personalized engagement strategies that can cope with the high individual variability of the targeted users.

## Supporting Information

S1 Data Data for figures and tables. This zip archive contains the data behind all figures and tables in CSV format.
(ZIP)
